# ADA1-Driven Metabolic Refueling Enhances CAR T Cell Therapy for Solid Tumors

**DOI:** 10.3390/cancers18010034

**Published:** 2025-12-22

**Authors:** Alex Wade Song, Xiaotong Song

**Affiliations:** 1 Cellula BioPharma, Inc., Houston, TX 77021, USA; info@cellula-biopharma.com; 2 Department of Translational Medical Sciences, College of Medicine, Texas A&M University, 2121 W Holcombe Blvd, Houston, TX 77030, USA; 3 Center for Infectious and Inflammatory Diseases, Institute of Bioscience and Technology, Texas A&M University, 2121 W Holcombe Blvd, Houston, TX 77030, USA

**Keywords:** CAR T cells, tumor microenvironment, metabolic reprogramming, adenosine, inosine, adenosine deaminase

## Abstract

CAR T cell therapy, while highly effective for hematological malignancies, continues to face significant hurdles in the treatment of solid tumors. Key challenges include severe nutrient deprivation and the presence of immunosuppressive metabolites such as adenosine in the tumor microenvironment, which limit CAR T cell persistence and antitumor activity. This review focuses on current progress and future directions for ADA1-based metabolic reprogramming as a targeted approach to enhance CAR T cell function. We discuss recent advances, particularly the engineering of CAR T cells to express ADA1, which facilitates the local conversion of immunosuppressive adenosine into inosine, thereby supporting T cell metabolism and improving therapeutic outcomes. Preclinical studies, including our own, demonstrate that ADA1-expressing CAR T cells exhibit reduced exhaustion, greater metabolic flexibility, and enhanced antitumor efficacy in solid tumor models. The selective clearance of adenosine and supplementation of inosine directly address the metabolic barriers within the tumor microenvironment and provide an effective strategy to bolster CAR T cell responses. Integration of ADA1-driven metabolic refueling with future innovations in CAR design holds promise for overcoming key obstacles in solid tumor immunotherapy. We conclude by highlighting the potential of ADA1-based strategies and offering our perspective on their translation toward clinical application.

## Simple Summary

Solid tumors create a metabolically hostile environment that limits chimeric antigen receptor (CAR) T cell persistence and function, in part through elevated adenosine and restricted nutrient availability. This review focuses on the selective metabolic reprogramming of CAR T cells by adenosine deaminase 1 (ADA1), which converts immunosuppressive adenosine to inosine, providing an alternative fuel and improving cell survival and migration. Engineered expression of ADA1 markedly boosts CAR T cell efficacy in preclinical models by overcoming nutrient competition and suppression, representing a promising direction for next-generation cellular therapies in solid malignancies.

## 1. Introduction

CAR T cell therapy has marked a major advancement in oncology, especially for patients with refractory B cell hematologic malignancies [1,2,3,4,5,6,7,8]. Pivotal clinical studies have shown that CAR T cell therapy can induce remission rates of 70–90% in acute lymphoblastic leukemia and diffuse large B cell lymphoma, resulting in multiple U.S. Food and Drug Administration (FDA)-approved CAR T cell products in recent years. This achievement highlights the powerful potential of genetic engineering to harness the immune system against cancer.

Nevertheless, hematologic cancers represent a relatively small part of the overall global cancer burden. Latest data from the Global Cancer Statistics (GLOBOCAN 2020) report estimates approximately 1.5 million new cases of leukemia, lymphoma, and multiple myeloma combined per year, accounting for less than 10% of all cancer cases and deaths globally [9]. In stark contrast, solid tumors—including lung, breast, colorectal, prostate, gastric, and liver cancers—account for the overwhelming majority, with more than 16 million new cases and over 8 million deaths reported annually [9]. For example, lung and breast cancers together were responsible for about 4.6 million new cases and nearly 2.4 million deaths worldwide in 2020. Overcoming the challenge of treating these solid malignancies is therefore essential to impact global cancer outcomes.

Despite clear principles of antigen targeting and cytotoxicity, the remarkable success of CAR T cell therapy in blood cancers has not translated well to solid tumors [10,11,12]. CAR T cells must contend with the unique barriers of the solid tumor microenvironment [13,14,15,16], including dense fibrotic stroma that restricts immune cell infiltration, significant antigenic variability leading to immune evasion, and a profoundly immunosuppressive and metabolically hostile milieu. These hurdles severely limit CAR T cell persistence and efficacy in solid cancers.

Given the enormous burden and mortality associated with solid tumors worldwide, it is crucial to understand and address these obstacles. Recent research has highlighted metabolic reprogramming as a promising strategy to overcome these challenges [14,17,18,19,20,21,22,23,24]. Rather than relying solely on conventional metabolic pathways such as glycolysis and oxidative phosphorylation, innovative approaches targeting key metabolites can enhance T cell fitness in adverse conditions. In particular, ADA1-mediated refueling presents a novel method for improving CAR T cell survival and antitumor activity [19,20,21,25,26,27]. By converting immunosuppressive adenosine to inosine, ADA1 extends the metabolic flexibility of CAR T cells, supporting their function when conventional nutrients are limited and reducing their susceptibility to exhaustion. This review focuses on the role of ADA1 in metabolic reprogramming strategies for CAR T cell therapy, summarizing recent advances and discussing the potential of selective inosine supplementation for solid tumor treatment.

## 2. Current Perspectives and Recent Advances in CAR T Cell Therapy for Solid Tumors

CAR T cell therapy has demonstrated impressive efficacy against hematological malignancies, but its translation to solid tumors faces unique hurdles, including antigen heterogeneity, immunosuppressive microenvironments, T cell exhaustion, and metabolic restrictions [13,28,29,30]. Recent innovations across several domains are laying the groundwork for improved outcomes.

### 2.1. Multi-Targeted CAR Designs

Antigen escape remains a major challenge for CAR T therapy in solid tumors. To mitigate this, multi-specific CAR architectures have been developed. Tandem CARs and dual CARs encode antigen-binding domains for two or more antigens, thereby reducing the likelihood of tumor escape and enhancing cytotoxicity [31,32]. Universal CAR systems that utilize adapter molecules for flexible targeting have also emerged, allowing for rapid switching between different tumor antigens without re-engineering CAR T cells [33,34]. These designs are showing promise in preclinical models and early-phase clinical trials.

### 2.2. Overcoming Immune Suppression

The solid tumor microenvironment (TME) is highly immunosuppressive, characterized by regulatory cell populations, inhibitory checkpoint molecules, and suppressive cytokines. To counter these, CAR T cells have been engineered to resist tumor-derived inhibitory signals, such as programmed cell death protein 1 (PD-1) and transforming growth factor beta (TGF-β) [35,36]. Combination therapies, where CAR T cells are administered alongside immune checkpoint inhibitors (ICIs), have enhanced persistence and antitumor activity in models of solid cancer [37,38]. Moreover, “armored” CAR T cells engineered to secrete cytokines like IL-15 or express costimulatory ligands are demonstrating improved survival and functionality [39].

### 2.3. Strategies to Address T Cell Exhaustion and Senescence

Chronic antigen exposure in the TME leads to T cell exhaustion, characterized by reduced effector function and increased expression of inhibitory receptors (PD-1, Lymphocyte-activation gene 3/LAG-3, T cell immunoglobulin and mucin-domain containing-3/TIM-3, cytotoxic T lymphocyte-associated protein 4/CTLA-4) [40]. Gene editing approaches have been used to knock out exhaustion markers or overexpress costimulatory molecules such as 4-1BB (also known as CD137; the full name is Tumor necrosis factor receptor superfamily member 9) and OX40 (also known as CD134; Tumor necrosis factor receptor superfamily member 4), enhancing CAR T cell persistence [41,42]. Interventions that influence key transcriptional regulators (thymocyte selection-associated high mobility group box/TOX, basic leucine zipper transcription factor/BATF) are being explored to reverse exhaustion and delay senescence [43,44].

### 2.4. Metabolic Reprogramming Approaches

Metabolic constraints, including hypoxia, competition for nutrients, and build-up of toxic metabolites such as adenosine, limit CAR T cell activity in solid tumors [19]. Engineering CAR T cells to overexpress enzymes like ADA or utilize alternative metabolic pathways (e.g., increased fatty acid oxidation, improved mitochondrial biogenesis) has yielded positive results [19,20,27]. Such metabolic reprogramming can be further optimized by in vivo interventions, such as combining CAR T cells with drugs that modulate the tumor metabolism.

## 3. The Tumor Microenvironment, Metabolic Barriers, and T Cell States

### 3.1. Hostile Features of the Solid Tumor Microenvironment

The solid TME is a complex, dynamic, and profoundly hostile milieu that actively impedes effective T cell-mediated immunity. Several key features define the TME, each exerting unique and mostly suppressive influences on infiltrating immune cells. Among these are chronic hypoxia, nutrient deprivation, high interstitial pressure, acidosis (resulting from excess lactic acid and poor perfusion), physical barriers such as dense extracellular matrix, and a wide array of immunosuppressive cytokines, metabolites, and regulatory cell populations.

### 3.2. Metabolic Competition and Suppression by Tumor Cells

Tumor cells rewire their metabolism to support unchecked proliferation, favoring aerobic glycolysis (the Warburg effect) over oxidative phosphorylation even in the presence of oxygen [45,46,47,48,49,50]. This metabolic reprogramming allows for the rapid generation of adenosine triphosphate (ATP) and anabolic precursors but also results in aggressive consumption of glucose and amino acids from the local environment. As a consequence, tumor-infiltrating T cells face severe shortages of glucose, glutamine, serine, and other key nutrients. In parallel, high rates of glycolysis and mitochondrial dysfunction in tumors result in increased secretion of lactate and hydrogen ions, further acidifying the extracellular space and hindering T cell viability and effector function.

### 3.3. Adenosine (ADO) as a Dominant Immunosuppressive Metabolite

A prominent immunosuppressive mechanism in the TME involves the accumulation of extracellular adenosine [51,52,53]. Under conditions of cellular stress, hypoxia, and inflammation—ubiquitous in tumors—large amounts of ATP are released into the extracellular compartment. This ATP is rapidly hydrolyzed by the ectonucleotidases CD39 and CD73, which are upregulated on tumor cells, regulatory T cells, stromal elements, and myeloid-derived suppressor cells. The concerted action of CD39 (converting ATP to adenosine monophosphate/AMP) and CD73 (AMP to adenosine) leads to a marked increase in extracellular adenosine levels. These can reach up to 100–200 μM in tumors, compared to sub-micromolar concentrations in healthy tissue. Adenosine, acting primarily through the high-affinity A2A receptor on T cells (with additional roles for A2B), profoundly suppresses T cell activation, proliferation, cytokine production, and cytotoxicity by elevating intracellular cAMP and interfering with T cell receptor (TCR) signaling pathways. Chronic exposure fosters an exhausted T cell phenotype and impairs immune memory, making the TME a nearly insurmountable barrier for naturally occurring and adoptively transferred T cells—including CAR T cells.

### 3.4. T Cell Metabolic States and Vulnerabilities in the TME (Table 1)

The impact of these metabolic constraints and immunosuppressive metabolites on T cells depends heavily on their differentiation and activation state:

• Naïve T cells rely on oxidative phosphorylation (OXPHOS) and fatty acid oxidation for homeostatic survival and are generally quiescent, making them less equipped to thrive in the inflammatory, nutrient-poor tumor bed [15,54].
• Upon activation, T cells undergo a metabolic switch to aerobic glycolysis, supporting intense proliferation and biosynthetic activity needed for effector functions. However, the high glycolytic demand cannot be met in the TME, resulting in suboptimal activation and rapid dysfunction [15,54].
• Memory T cells, including central (T_CM) and effector (T_EM) subsets, possess enhanced mitochondrial mass and metabolic plasticity, allowing more effective adaptation in hostile environments. Nevertheless, they remain susceptible to inhibition by adenosine, lactic acid, and nutrient deprivation [55,56,57].
• Exhausted T cells, which accumulate in tumors after chronic antigen exposure and ongoing metabolic stress, are characterized by impaired mitochondrial function, low energy reserves, diminished glycolytic and oxidative capacity, and sustained expression of inhibitory receptors such as PD-1, LAG-3, and TIM-3. They show reduced proliferation, cytokine secretion, and cytolytic activity, further reinforced by high adenosine via the CD39/CD73 axis [58,59,60]. This ultimately promotes apoptosis and loss of potentially tumor-reactive clones.

**Table 1:** Metabolic profiles, functional outcomes, and key cell surface receptors of T cell subsets.

T Cell Subset	Dominant Metabolic Program	Metabolic Features	Functional Role in Immunity	Key Surface Receptors
Naïve T Cell [15,54]	OXPHOS, fatty acid oxidation (FAO)	Low nutrient uptake, energy-efficient, quiescent, high AMPK and low mTOR activity	Long-term surveillance, maintenance of diversity	CD45RA, CD62L, CCR7, TCR (low activation)
Activated Effector T Cell [15,54]	Aerobic glycolysis, increased glutaminolysis	High nutrient (glucose, glutamine) uptake, strong anabolic drive, high mTOR activity, rapid biosynthesis	Proliferation, cytokine secretion, cancer cell killing	CD25 (IL-2Rα), CD28, GLUT1, CD98, CD69, TCR (high activation)
Central Memory T Cell [55,56,57]	OXPHOS, FAO, preserved glycolytic capacity	Mitochondrial remodeling, increased spare respiratory capacity, energy flexibility	Long-term survival, rapid reactivation, migration	CD45RO, CD62L, CCR7, IL-7Rα (CD127), TCR
Effector Memory T Cell [55,56,57]	Mixed OXPHOS and glycolysis	Intermediate metabolic activity, poised for effector function	Immediate protection at peripheral tissues	CD45RO, lower CD62L, CXCR3, TCR
Exhausted/Dysfunctional T Cell [58,59,60]	Impaired glycolysis and OXPHOS, ADO accumulation, disrupted mTOR signaling	Mitochondrial fragmentation, low ATP production, bioenergetic crisis, upregulation of inhibitory pathways	Dysfunction, poor proliferation, loss of cytotoxicity	PD-1, TIM-3, LAG-3, CTLA-4, A2A adenosine receptor

## 4. Extracellular Adenosine: Production, Accumulation, and Immunosuppressive Functions

Extracellular adenosine is a central mediator of immunosuppression in solid tumors, with profound effects on T cell function, as well as direct contributions to cancer cell survival and growth. Its importance in the TME stems from a well-orchestrated process of production and accumulation, coupled with a complex network of signaling and metabolic consequences.

### 4.1. Mechanisms of Adenosine Generation: The CD39/CD73 Axis

Under physiologic conditions, extracellular adenosine concentrations remain low; however, the hypoxic, stressed environment of solid tumors leads to a dramatic upregulation in adenosine production. This is largely due to increased expression and activity of two key ectoenzymes, CD39 (ENTPD1) and CD73 (NT5E), on the surface of tumor cells, cancer-associated fibroblasts, endothelial cells, regulatory T cells (Tregs), and certain myeloid subsets [61,62,63,64,65].

The production cascade begins when damaged or stressed cells in the TME release large amounts of ATP and ADP as “danger signals”. CD39 catalyzes the sequential hydrolysis of these nucleotides, from ATP/ADP (adenosine diphosphate) to AMP:CD39; removes phosphate groups from ATP and ADP, converting them into AMP; and from AMP to adenosine: CD73, anchored to the cellular membrane, then hydrolyzes AMP to adenosine [61,62].

Hypoxia-inducible factors (HIFs), which accumulate in response to low oxygen tension—a universal trait of solid tumors—directly upregulate the transcription of both CD39 and CD73, further increasing the capacity for adenosine generation. In advanced tumors, high densities of CD39+CD73+ cells can lead to adenosine concentrations in the TME on the order of hundreds of micromolar, several-fold higher than in normal tissue environments [13,61,62,66,67].

### 4.2. Immunosuppressive Signaling via A2A and A2B Receptors on T Cells

Once generated, adenosine exerts profound regulatory effects on immune cell function primarily through binding to specific G protein-coupled receptors. Four adenosine receptor subtypes exist (A1, A2A, A2B, and A3), but A2A and A2B are the most relevant in the context of immunosuppression. Both are expressed on T cells and NK cells; however, A2A has especially high affinity and is the dominant immunoregulatory receptor under the high adenosine levels seen in tumors.

Upon adenosine binding, A2A receptors activate the Gs protein, stimulating adenylate cyclase and increasing intracellular cAMP [68,69,70,71,72,73,74,75]. This signaling cascade:

• Inhibits proximal TCR signaling (by interference with protein kinase C, Zap70/Zeta-chain (TCR)-associated protein kinase 70, and downstream NFAT and NF-κB activation);
• Suppresses cell proliferation and blockades cell cycle progression of T cells;
• Reduces cytotoxic function by lowering the expression of perforin and granzymes and weakening immune synapse formation;
• Blocks cytokine gene expression and secretion (notably IFN-γ/interferon gamma, IL-2, TNF-α/tumor necrosis factor alpha) even in already activated cells, thereby blunting further immune recruitment and amplification;
• Promotes T cell exhaustion by enhancing the expression of co-inhibitory receptors (PD-1, TIM-3, LAG-3) and reducing metabolic fitness, ultimately predisposing cells to apoptosis.

A2B receptors, which have lower affinity for adenosine but are upregulated under hypoxic and inflammatory conditions, further promote immunosuppression via effects on dendritic cells, macrophages, and regulatory T cells, driving them toward tolerogenic phenotypes [73,74,76,77]. Importantly, chronic exposure to high adenosine not only functionally incapacitates effector cells but also blocks the priming of new antitumor responses.

### 4.3. Adenosine as a Metabolic Modulator Supporting Cancer Cell Survival and Progression

Beyond its impact on immune cells, adenosine acts as a paracrine and autocrine growth factor for cancer and stromal cells through both A2A and especially A2B receptor signaling:

• Stimulation of glycolysis: Adenosine-A2B engagement promotes glycolytic metabolism in cancer cells, supporting rapid proliferation under low-oxygen conditions [73,74,76,77,78,79,80].
• Induction of angiogenesis: A2B receptor signaling upregulates the expression of VEGF and other pro-angiogenic factors, enhancing the blood supply to the tumor [78].
• Facilitation of immune evasion: Adenosine shapes the TME by promoting the expansion and suppressive activity of regulatory T cells (Tregs) and myeloid-derived suppressor cells (MDSCs), which further reinforce immunosuppressive and pro-tumorigenic conditions [69,75,76,81,82].
• Resistance to therapy: Adenosine-rich environments have been linked to resistance to immune checkpoint blockade, chemotherapy, and radiation, largely due to protected “immune-privileged” metabolic niches that shield cancer cells from immune elimination [83].

Thus, adenosine accumulation is not merely a byproduct of tumor growth but serves as a fundamental regulator of both tumor cell and immune cell fate. The CD39/CD73-mediated adenosine axis exemplifies the integration of metabolic, vascular, and immunoregulatory control within the TME, making it a compelling target for therapeutic intervention and metabolic reprogramming strategies in next-generation CAR T cell therapy.

## 5. ADA1-Mediated Metabolic Refueling in CAR T Cells

### 5.1. Mechanistic Basis of ADA1 Function in T Cells

ADA1 is an essential enzyme in purine metabolism that catalyzes the irreversible deamination of adenosine to inosine [61,84,85]. While ADA1 is widely expressed in lymphoid tissues, its dynamic functional role within tumor-infiltrating lymphocytes—particularly CAR T cells—has only recently come into focus. In the solid tumor microenvironment, adenosine accumulates due to upregulated ectonucleotidases (such as CD39 and CD73), driving profound suppression of T cell function via A2A receptor engagement [61,62,64,65,68,73,77,79,83,86,87,88,89,90,91,92]. This suppressive axis leads to reduced T cell activation, cytokine production, and eventually, exhaustion.

Engineering CAR T cells to overexpress ADA1 offers a mechanistically distinct pathway to mitigate adenosine toxicity. By converting immunosuppressive adenosine into inosine, ADA1 disrupts the suppressive signals and simultaneously generates a metabolically useful substrate. Inosine, unlike adenosine, does not strongly activate T cell inhibitory receptors, and it can serve as an alternative carbon source, entering glycolytic and pentose phosphate pathways to support energy generation and biosynthesis under conditions of glucose restriction [25,93]. Recent studies have confirmed that ADA1-overexpressing T cells not only resist adenosine-induced suppression but also shift their metabolic program toward greater flexibility and robustness in hostile environments.

**Figure 1:**
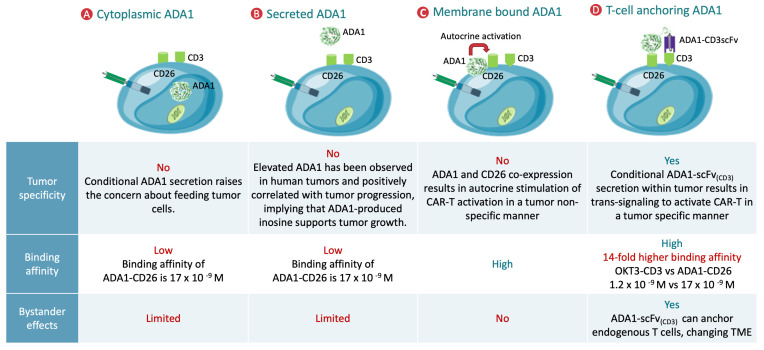
Strategies for ADA1 localization in CAR T cells: impact on tumor specificity, binding affinity, and bystander effects. Schematic comparison of four ADA1 (adenosine deaminase 1) localization strategies in CAR T cells and their functional implications: (**A**) Cytoplasmic ADA1: ADA1 is retained intracellularly with minimal impact on the local tumor microenvironment (TME). Conditional secretion risks non-specific benefits to tumor cells and lacks tumor specificity, binding affinity, and bystander activation. (**B**) Secreted ADA1: ADA1 is broadly secreted into the extracellular space, mimicking effects seen in some human tumors, where elevated ADA1 is associated with tumor progression due to inosine support for cancer cell growth. This approach lacks tumor specificity and high binding affinity, and has limited bystander effects. (**C**) Membrane-bound ADA1: ADA1 is tethered to the T cell surface via CD26, promoting high binding affinity but causing non-specific autocrine stimulation of CAR T cells, regardless of tumor presence, thus lacking tumor specificity and robust bystander engagement. (**D**) T cell-anchored ADA1 (ADA1-scFvCD3): ADA1 is anchored at the T cell surface using a tumor-activated scFv targeting CD3 (ADA1-scFv(CD3)). This strategy provides tumor-restricted ADA1 activity, significantly increases local binding affinity, and enables bystander effects by anchoring endogenous T cells and remodeling the TME. Conditional ADA1-scFv(CD3) secretion within tumors enables trans-signaling for selective CAR T activation and improved safety and efficacy.

Various strategies for localizing ADA1 in CAR T cells have important implications for tumor specificity, binding affinity, and effects within the tumor microenvironment. As illustrated (Figure 1) [19,20,21,25,26,27], cytoplasmic or secreted ADA1 can lead to non-specific distribution, raising concerns about inadvertently supporting tumor cell metabolism or lacking precise targeting, and both approaches offer only limited functional benefits. Membrane-bound ADA1, typically anchored via CD26, improves binding affinity but may result in tumor non-specific, autocrine activation of CAR T cells, which could compromise safety or therapeutic selectivity. In contrast, the engineered approach of anchoring ADA1 to T cells through a tumor-activated scFv (ADA1-scFv(CD3)) provides several distinct advantages: enhanced tumor specificity, dramatically increased local binding affinity, and the ability to recruit and activate endogenous T cells via bystander effects. This strategy conditions ADA1 activity to the tumor site, thereby maximizing antitumor efficacy while minimizing systemic or off-target risks, and facilitates broader immune remodeling of the tumor microenvironment.

### 5.2. Synergistic Role of CD26 and ADA1 in Metabolic Reprogramming

CD26—also known as dipeptidyl peptidase IV (DPP4)—is a multifunctional type II transmembrane glycoprotein widely expressed on the surface of several cell types, including activated T cells [94,95,96,97,98,99]. CD26 possesses exopeptidase activity, cleaving dipeptides from the amino terminus of polypeptides, and serves key roles in immune regulation, T cell activation, and modulation of chemokine and cytokine activity. It has been implicated in the regulation of T cell costimulatory signaling, influencing both T cell proliferation and effector function, and can participate in adhesion and migration by interacting with ADA1, caveolin-1, and the extracellular matrix.

Significantly, CD26 acts as the primary anchoring receptor for ADA1 on the T cell plasma membrane [84,100,101,102,103,104,105,106]. ADA1, an essential enzyme in purine metabolism, catalyzes the deamination of adenosine to inosine, thereby reducing extracellular adenosine concentrations. This interaction between CD26 and ADA1 is both physical and functional: cell-surface CD26 binds ADA1, effectively immobilizing the enzyme in the pericellular region where adenosine builds up, especially in the immunosuppressive tumor microenvironment. This localization dramatically enhances the efficiency of adenosine clearance exactly where the immunosuppressive burden is highest.

**Figure 2:**
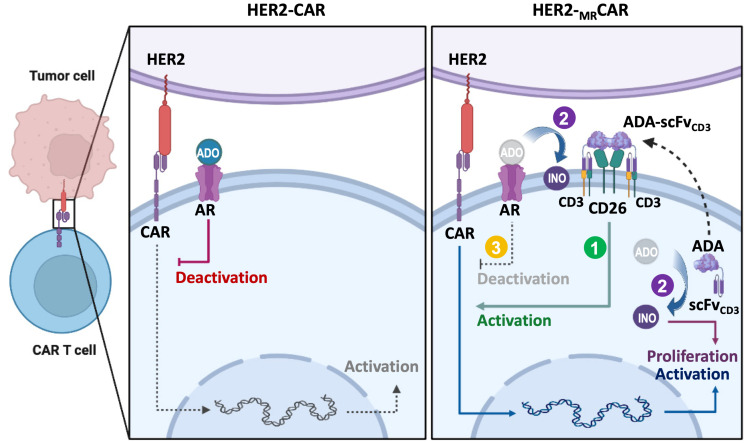
Multifunctional enhancement of CAR T cells by CD26/ADA1 coexpression. Schematic representation of MRCAR-T cells engineered to express both a tumor-specific chimeric antigen receptor (CAR) and CD26/ADA1 (Adenosine deaminase 1) via the MR-vector system. (1) CD26 enhances CAR T cell trafficking and infiltration into bulky tumor masses, while (2) ADA1 enzymatically converts adenosine (ADO) to inosine (INO), providing an alternative carbon source for CAR T cell proliferaion and metabolic support. (3) ADA also alleviate adenosine-mediated immunosuppression within the tumor microenvironment. The coordinated actions of CD26 and ADA1 collectively boost CAR T cell persistence, cytotoxicity, and antitumor efficacy against solid tumors.

In the context of CAR T cell metabolic engineering, coexpression of CD26 and ADA1 ensures high-level, membrane-bound ADA1 enzymatic activity (Figure 2). This optimally positions ADA1 to degrade adenosine at the immune synapse or immediate tumor-immune interface, supporting T cell activation, cytokine production, and antitumor functions. Thus, CD26 is more than a metabolic or costimulatory molecule—it is essential for the spatial regulation of ADA1 activity in engineered CAR T cells, maximizing the therapeutic advantage by enabling local adenosine detoxification and enhanced metabolic support.

### 5.3. Impact of ADA1-Mediated Refueling on CAR T Cell Phenotype and Function

Multiple in vitro and in vivo studies have demonstrated that ADA1-mediated metabolic refueling confers profound functional advantages to CAR T cells. Firstly, ADA1 expression reduces apoptotic and necrotic cell death under metabolic stress, supporting greater overall cell viability. Secondly, CAR T cells with enhanced ADA1 activity exhibit improved proliferation rates even under conditions of glucose scarcity or elevated adenosine, likely attributable to their ability to efficiently utilize inosine.

Perhaps most notably, ADA1-driven refueling alters T cell differentiation and memory formation [19,20,21,25,26,27]. Engineered CAR T cells exposed to high inosine concentrations during activation display increased expression of Tcm and stem cell memory (Tscm) markers, while exhibiting reduced levels of exhaustion-associated molecules such as PD-1, TIM-3, and LAG-3. In vivo, these phenotypic shifts correspond to better persistence, augmented migration into tumor tissues, and greater resistance to TGF-β-mediated suppression. The net result is superior tumor control compared to conventional CAR T cells.

### 5.4. Preclinical Models and Translational Relevance

Preclinical assessment of ADA1/CAR T cell constructs has been undertaken in several mouse tumor models, including hepatocellular carcinoma and non-small cell lung cancer. These studies consistently report that ADA1-engineered CAR T cells are able to reduce tumor volume more efficiently and persist longer in treated animals. Mechanistically, this improvement correlates with enhanced survival, greater infiltration into tumor parenchyma, and maintained effector functions despite the hostile metabolic microenvironment.

Importantly, these models provide early insights into safety and specificity. ADA1-expressing CAR T cells show no evidence of off-target toxicity or unwanted fueling of tumor cells, particularly when fusion constructs and cell surface localization strategies are used. These findings suggest that ADA1/CAR T metabolic engineering could be compatible with existing clinical protocols and combination treatments, subject to further validation.

## 6. Challenges, Controversies, and Future Directions in ADA1-Mediated CAR T Cell Metabolic Reprogramming

### 6.1. Technical, Biological, and Translational Challenges

The emergence of ADA1-mediated metabolic refueling for CAR T cells in solid tumors marks a significant advance in the field of cellular immunotherapy. However, multiple technical and biological challenges warrant careful consideration for its clinical translation. Achieving stable and controlled ADA1 expression in CAR T cells remains nontrivial, as vector design, cellular source, and manufacturing protocols can influence enzymatic activity and functional persistence. Overexpression may lead to off-target effects, altered purine metabolism, or unintended immune activation. Conversely, suboptimal ADA1 levels may be insufficient to overcome the high concentrations of adenosine typically found in the tumor microenvironment.

A major biological challenge lies in the complexity and heterogeneity of the solid tumor microenvironment. Factors such as nutrient deprivation, variable adenosine production, and the presence of suppressive stromal and immune cells differ not only between tumor types but also between patients and even regions within a tumor. For instance, while ADA1-engineered CAR T cells may thrive in adenosine-rich cancers such as hepatocellular carcinoma, their efficacy in tumors with alternative dominant suppressive pathways remains uncertain. Thus, patient stratification and personalized engineering will likely be necessary for optimal application.

One of the central challenges in advancing ADA1-mediated CAR T cell metabolic reprogramming is the intricate complexity of the TME, which is not fully recapitulated by traditional in vitro or even animal models. The development of customized microfluidic platforms—so-called “tumor-on-a-chip” systems—offers a promising avenue to address this limitation [107,108,109]. These platforms allow precise control over spatial gradients of nutrients, oxygen, and immunosuppressive metabolites, and can reproduce key structural and mechanical features of the TME. By incorporating multiple cell types (tumor, stromal, endothelial, and immune cells) and dynamic flow conditions, microfluidic models provide a more physiologically relevant setting to evaluate CAR T cell infiltration, survival, and function under conditions that closely mimic the in vivo TME. Importantly, these systems can be used to systematically test and optimize ADA1-based or other metabolic interventions, assess potential off-target effects, and even personalize therapy by using patient-derived tumor cells. Thus, the integration of microfluidic modeling represents an important step toward improving translational relevance and predictive power in the preclinical development of next-generation CAR T cell therapies.

In standard hematologic CAR T cell therapy, the most characteristic and serious adverse effects are cytokine release syndrome (CRS) and immune effector cell-associated neurotoxicity syndrome (ICANS), both of which are linked to heightened systemic cytokine production and off-tumor, off-target activation [110,111,112,113,114]. In the context of preclinical ADA1-CAR T cell studies, available data suggest that ADA1-mediated adenosine metabolism does not exacerbate, and may in some settings attenuate, excessive cytokine production compared to conventional CAR T cell therapies. For example, in non-small cell lung cancer (NSCLC) and hepatocellular carcinoma (HCC) syngeneic mouse models, Song et al. observed improved tumor control with ADA1/CAR T cells, along with evidence of decreased T cell exhaustion and preservation of long-term function, but without significant elevations in serum pro-inflammatory cytokines or indicators of systemic toxicity [19]. This suggests that metabolic refueling via ADA1 may promote a more sustainable, less hyperactivated T cell phenotype, potentially reducing the risk of CRS or neurotoxicity. However, these findings must be interpreted with caution, as mouse models do not fully recapitulate the complexity of human immune responses, and interspecies differences can obscure certain toxicities.

Comparatively, clinical experience with CAR T cell therapy in humans underscores the need for careful monitoring and management of these immune-related adverse effects, with CRS rates reported as high as 70–90% in some cohorts, and neurotoxicity in 30–50% of cases [110,111,112,113,114]. While preclinical ADA1 studies are promising from a safety standpoint, broader issues—such as the risk of ectopic adenosine depletion, potential for unintended tissue damage, or metabolic disturbances—require further evaluation during clinical translation. To date, there are no clinical trials of ADA1/CAR T cells in humans, and the true safety profile can only be fully ascertained through phase I clinical evaluation. Future studies will need to closely monitor for both known CAR T cell toxicities and any unique on- or off-target effects associated with metabolic pathway modulation.

### 6.2. Controversies and Knowledge Gaps in Metabolite Manipulation

Beyond technical hurdles, controversies exist regarding the broader ramifications of manipulating purine metabolism in the tumor microenvironment. While ADA1 converts immunosuppressive adenosine into inosine, emerging evidence suggests that certain tumor cells may metabolize inosine to fuel their own proliferation under nutrient stress [61]. The net effect—whether ADA1 refueling inadvertently supports tumor growth or predominantly benefits engineered T cells—remains under active investigation. Designing ADA1 constructs that preferentially localize inosine production to CAR T cells or restrict its accessibility to tumor cells is a promising area, but further comparative studies across cancer models are critically needed.

Another point of debate concerns the interplay between metabolic reprogramming, T cell exhaustion, and memory phenotype formation. Although ADA1 activity is associated with improved central memory and stem cell memory profiles in preclinical models [81,115], the mechanistic underpinnings are not fully delineated. It is unclear whether ADA1 directly influences epigenetic states, signaling cascades, or metabolic checkpoints responsible for durable T cell function. Multi-omics approaches, longitudinal tracking, and single-cell technologies are required to dissect these processes and guide rational engineering efforts.

### 6.3. Comparative Perspectives and Need for Integrated Strategies

ADA1-mediated metabolic engineering offers notable specificity compared with broader interventions such as glycolytic enhancement, mitochondrial optimization, or amino acid supplementation. However, current evidence suggests that single-modality strategies may not suffice to counteract the diverse and adaptive landscape of the solid TME. Combining ADA1 engineering with additional metabolic, immunomodulatory, or homing technologies—such as coexpression of chemokine receptors, checkpoint blockade, or cytokine tuning—could yield synergistic benefits [38,116]. Defining the optimal configuration and timing for these combinatorial therapies remains a priority for translational research.

### 6.4. Clinical Translation and Regulatory Considerations

Moving ADA1-mediated CAR T cells towards clinical practice demands a rigorous evaluation of safety, efficacy, and scalability. Long-term persistence of engineered T cells, avoidance of unintended autoimmunity, and minimization of systemic metabolic disturbances are paramount concerns. Robust preclinical models—including humanized mice and ex vivo tissue systems—should be leveraged to assess toxicity, efficacy in immunocompetent hosts, and the risk of off-target inosine fueling. In parallel, standardized protocols for manufacturing, quality control, and analytical monitoring must be developed in partnership with regulatory agencies to address the unique challenges posed by metabolic gene therapy.

Another key translational need is the establishment of reliable biomarkers to predict and monitor ADA1/CAR T cell activity and patient response [117,118,119]. Measuring systemic and intratumoral levels of adenosine, inosine, exhaustion markers, and memory phenotypes before and after therapy could facilitate patient selection and enable adaptive clinical trial designs. Integration with non-invasive imaging and liquid biopsy approaches will further enhance the clinical toolkit.

### 6.5. Directions for Future Research and Reconceptualization

Despite notable progress, the scientific understanding of metabolic reprogramming—particularly ADA1-centric manipulation—remains incomplete. Future research should address several critical questions:

• Mechanistic Elucidation: How does ADA1 activity reshape metabolic pathways, signaling networks, and epigenetic landscapes within CAR T cells? What are the regulatory circuits that link inosine utilization to memory formation and exhaustion resistance?
• Tumor Heterogeneity and Resistance: How do distinct tumor types, metabolic niches, and adaptive responses influence ADA1/CAR T cell efficacy and long-term durability? Can signatures of TME composition be harnessed for personalized therapy?
• Safety Optimization: What are the risks of inadvertent tumor support, autoimmune responses, or metabolic imbalances arising from ADA1 activity? How can spatially and temporally controlled expression systems, such as inducible or tissue-specific promoters, mitigate these risks?
• Clinical Integration: What are the best practices for combining ADA1-engineered CAR T cells with other immunotherapies, metabolic inhibitors, or emerging CART platforms? How can clinical trials be designed to capture the full impact of these multifaceted interventions?
• Broader Applicability: Can ADA1-mediated refueling be extended to other cell therapies, such as TCR-T cells, NK cells, or macrophage engineering? What are the implications for other metabolite-mediated immunosuppressive axes in cancer and beyond?

Additionally, integrating innovative diagnostic tools such as nanoscale imaging and high-throughput flow cytometry for liquid biopsies will enable real-time monitoring of CAR T dynamics and tumor response, supporting more precise and adaptive therapeutic strategies [120,121].

## 7. Conclusions

By addressing these challenges and knowledge gaps, the field of metabolic reprogramming will be poised not only to expand the reach of CAR T cell therapy in solid tumors but also to offer a new lens for immunotherapeutic innovation. The integration of metabolic engineering, systems biology, and clinical translation represents a promising reconceptualization of cancer immunotherapy—transforming how cellular therapies are designed, optimized, and personalized for the most intractable malignancies.

Looking ahead, future action lines should include deeper mechanistic studies of ADA1 activity and its effects on CAR T cell biology, development of advanced preclinical models that more accurately mimic the tumor microenvironment, and multi-modal clinical trials exploring combinations with other metabolic or immunotherapeutic interventions. The application of innovative platforms such as microfluidic systems, nanoscale imaging, and high-throughput liquid biopsy technologies will also be crucial for optimizing and individualizing treatment strategies. Through these multifaceted efforts, the promise of ADA1-driven metabolic reprogramming in CAR T cell therapy can be fully realized for patients facing refractory solid cancers.

## Data Availability

No new data were created or analyzed in this study. Data sharing is not applicable to this article.
